# Setup Error Differences and Margin Expansion Among Rectal Cancer Segments: Consistency Evaluation Between EPID and iSCOUT

**DOI:** 10.1002/cnr2.70515

**Published:** 2026-03-20

**Authors:** Bao Wan, Jiangtao Han, Qian Wu, Jingjing Lu, Xueshan Li, Yongtai Zheng, Chao Liu, Shuo Sun, Yu Zhao, Fukui Huan, Tantan Li

**Affiliations:** ^1^ Department of Radiation Oncology National Cancer Center/National Clinical Research Center for Cancer/Cancer Hospital, Chinese Academy of Medical Sciences and Peking Union Medical College Beijing China

**Keywords:** image‐guided, radiotherapy, rectal cancer, setup error

## Abstract

**Purpose:**

To analyze the differences in setup errors among patients with rectal cancer in the upper, middle and lower segments and evaluate the consistency between electronic portal imaging device (EPID) and iSCOUT image‐guided techniques.

**Methods:**

We retrospectively included 277 patients with rectal cancer treated with radiotherapy at our center between January 2020 and June 2025 and divided them into upper (*n* = 23), middle (*n* = 115) and lower (*n* = 139) rectal cancer groups based on their pathological results. Setup errors and corresponding planning target volume (PTV) margins were calculated and compared across groups and between EPID and iSCOUT, with correlation and agreement between the two image‐guidance methods further evaluated.

**Results:**

The required external margins (cm) in the left–right (LR, *X*), superior–inferior (SI, *Y*) and anterior–posterior (AP, *Z*) directions for patients with upper, middle and lower rectal cancers were (0.44, 0.83, 0.57), (0.50, 0.69, 0.59) and (0.43, 0.68, 0.53), respectively. The setup errors between the upper and lower rectal cancers in the *Z* direction (*p* = 0.013) were significantly different. The *X* and *Y* directions between the EPID and iSCOUT groups in the different segments of rectal cancer were significantly different. The registration results of the EPID in the *X*, *Y* and *Z* directions significantly correlated with the corresponding iSCOUT error data (*p* < 0.001). The 95% consistency limits of EPID and iSCOUT measurement results in the *X*, *Y* and *Z* were −3.65 to 4.38, −3.69 to 4.21 and −3.66 to 3.57 mm, respectively.

**Conclusion:**

In the *Y* direction, different margin expansions should be adopted based on the different rectal cancer treatment segments. The iSCOUT guidance technology can replace the EPID when necessary.

## 1 Introduction

Colorectal cancer is the third most common cancer and the second leading cause of cancer‐related deaths worldwide. Rectal cancer accounts for one‐third of these cases [1]. Recently, the overall incidence and mortality rates of colorectal cancer have gradually decreased. However, there is a trend of increasing incidence of rectal cancer in individuals < 50 years old [2, 3, 4]. Significant progress in rectal treatment has been made with the advent of total mesorectal excision and total neoadjuvant therapy. These treatment methods significantly reduced local recurrence rates and improved patient survival outcomes [5, 6, 7, 8]. Multidisciplinary combined treatments are commonly used for rectal cancer, and neoadjuvant chemoradiotherapy is becoming the standard treatment for locally advanced rectal cancer [9]. However, for patients with colorectal who have progressed to locally advanced stages, radical surgical resection is difficult and radiotherapy is an important treatment method [10, 11, 12].

According to the National Comprehensive Cancer Network (NCCN) and related guidelines, clinical radiotherapy for rectal cancer usually divides the rectum into the lower, middle and upper rectums based on the distance from the tumor to the anal verge or the peritoneal reflection determined by magnetic resonance imaging and computed tomography (CT) [13, 14]. Previous studies have investigated patient setup errors in rectal cancer radiotherapy. Xu et al. [15] reported that real‐time correction using a 6‐degree‐of‐freedom couch significantly reduced setup errors in all directions. Mohamed et al. [16] analyzed 189 patients and found that daily image‐guided radiotherapy (IGRT) reduced setup errors, recommending PTV margins of 0.7 cm for daily IGRT and 1.0 cm for non‐daily IGRT, with higher margins required for patients in the prone position (1.2 cm) or with BMI > 30 kg/m^2^ (1.4 cm). In addition, the rectum may change its position and shape due to factors such as bladder distension and body mass fluctuation [17]. However, current studies mostly report the rectum as a whole. A systematic analysis of the characteristics of setup errors in different rectal segments is lacking. Given the differences in the anatomical location and physiological mobility of these segments, it is of great significance to further detail the causes and differences of errors in each rectal cancer segment, which is expected to provide a scientific basis for clinical treatment decision‐making and the formulation of individualised radiotherapy plans. The iSCOUT image‐guided technology uses kilovoltage X‐ray stereoscopic planar imaging. It controls two X‐ray imaging units based on the isocenter point using registration software to obtain X‐ray projection images in two directions. Based on the patient's internal anatomical structural characteristics, two‐dimensional and three‐dimensional image registration was conducted between the X‐ray images and CT localisation images. This product can be used with radiotherapy to correct errors in patient setup.

In this study, we aimed to analyze the differences in setup errors among different rectal cancer patient groups based on the electronic portal imaging device (EPID) and iSCOUT setup error data generated within the same treatment fraction for each patient, providing a reference for target margin expansion in different rectal cancer segments. The correlation and consistency of the error data measured by EPID and iSCOUT in different rectal cancer segments were further assessed to evaluate the auxiliary role of iSCOUT in pretreatment position verification.

## 2 Materials and Methods

### 2.1 Case Selection and Inclusion Criteria

Patients with rectal cancer who received radiotherapy at the Cancer Hospital of the Chinese Academy of Medical Sciences between January 2020 and June 2025 were retrospectively included.

The following inclusion and exclusion criteria were applied to select patients for this study. Inclusion criteria: (1) Diagnosed with rectal cancer confirmed by pathology; (2) age 18 and 65 years; (3) Karnofsky performance status (KPS) score ≥ 70; (4) signed informed consent. Exclusion criteria were as follows: (1) severe dysfunction of major organs (heart, lung, liver, kidney) preventing completion of radiotherapy; (2) inability to complete the radiotherapy course due to voluntary withdrawal, psychological barriers or other study‐related reasons.

The patients were divided into three groups following their pathological diagnosis: upper, middle and lower groups. Patients with upper‐middle and middle‐lower rectal cancers were classified into the middle and lower rectum groups, respectively, following the relative position of the peritoneal reflection to the tumor.

Based on these criteria, a total of 277 patients were included, of whom 23 had upper rectal cancer (UR), 115 had middle rectal cancer (MR) and 139 had lower rectal cancer (LR). Table 1 shows the patients' clinical characteristics. We conducted 2443 EPID scans on 277 patients. The UR group (23 patients) underwent 203 EPID scans, the MR group (115 patients) underwent 1022 EPID scans and the LR group (139 patients) underwent 1218 EPID scans. This study received ethical approval from the hospital's ethics committee (approval no. 23/049‐3788). All patients provided written informed consent.

**TABLE 1:** Baseline characteristics of patients.

Characteristics	Upper‐rectum (*N* = 23)	Mid‐rectum (*N* = 115)	Lower‐rectum (*N* = 139)	H/χ2/F	*p*
Age (years), mean (range)	55.5 (30–72)	57.4 (33–81)	58.2 (15–83)	1.222	0.543
Gender					
Male	9	70	90	5.664	0.059
Female	14	44	48		
BMI, mean (SD)	23.6 (2.2)	23.6 (3.1)	23.4 (2.8)	0.185	0.832
*T* stage, *N* (%)					0.001
*T*1	0 (0)	1 (0.9)	4 (2.9)		
*T*2	1 (0)	5 (4.3)	13 (9.4)		
*T*3	7 (30.4)	76 (66.1)	105 (75.5)		
*T*4	13 (56.5)	33 (28.7)	17 (12.2)		
*Tx*	2 (8.7)	0 (0)	0 (0)		
*N* stage, *N* (%)					0.001
*N*0	1 (4.3)	9 (7.8)	26 (18.7)		
*N*1	4 (17.4)	40 (34.8)	58 (41.7)		
*N*2	16 (69.6)	64 (55.7)	47 (33.8)		
*Nx*	2 (8.7)	2 (1.7)	8 (5.8)		
*M* stage, *N* (%)					0.001
*M*0	1 (4.3)	111 (96.5)	129 (92.8)		
*M*1	20 (86.7)	2 (1.7)	6 (4.3)		
*Mx*	2 (8.7)	2 (1.7)	4 (2.9)		

Abbreviation: BMI, body mass index.

### 2.2 CT Simulation and Positioning Methods

**FIGURE 1:**
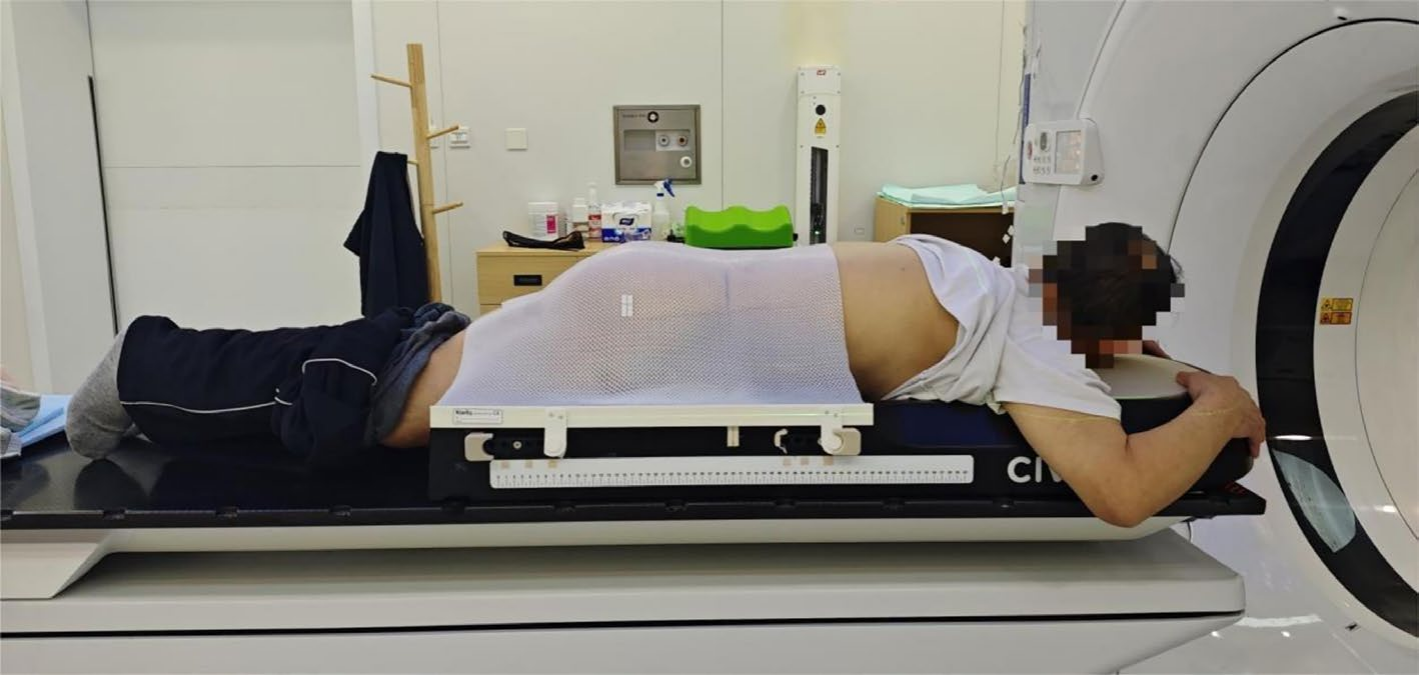
Postural fixation methods of the patient.

Before CT simulation, patients were asked to drink water to distend their bladders. All patients were fixed with an abdominopelvic frame and a prone positioning membrane, placed in the prone position with their head first. Their hands were folded around the front end of the abdominopelvic frame with quiet, free breathing. The thermoplastic membrane was placed in a constant‐temperature water bath for 15 min before positioning and then placed on the patient. CT images were acquired using a Brilliance CT Big Bore scanner (Philips, the Netherlands) to determine the isocenter position. The CT scan and reconstruction slice thicknesses were 5 mm. The scanned images were transferred to a pinnacle planning system (Philips, the Netherlands) for target and organ‐at‐risk delineation. The physicist designed the radiotherapy plan. Figure 1 shows the postural fixation methods of the patient.

### 2.3 Pre‐Treatment Preparation and Image Guidance

All patients underwent volumetric modulated arc therapy on a Varian accelerator (Varian, USA). Patients were asked to empty their bowels and bladder before each treatment and drink 800–1000 mL of water, as prescribed by the physician. Bladder distension was assessed by using a portable ultrasonography device. Radiotherapy was conducted if the requirements were met; otherwise, the preparation process was repeated.

**FIGURE 2:**
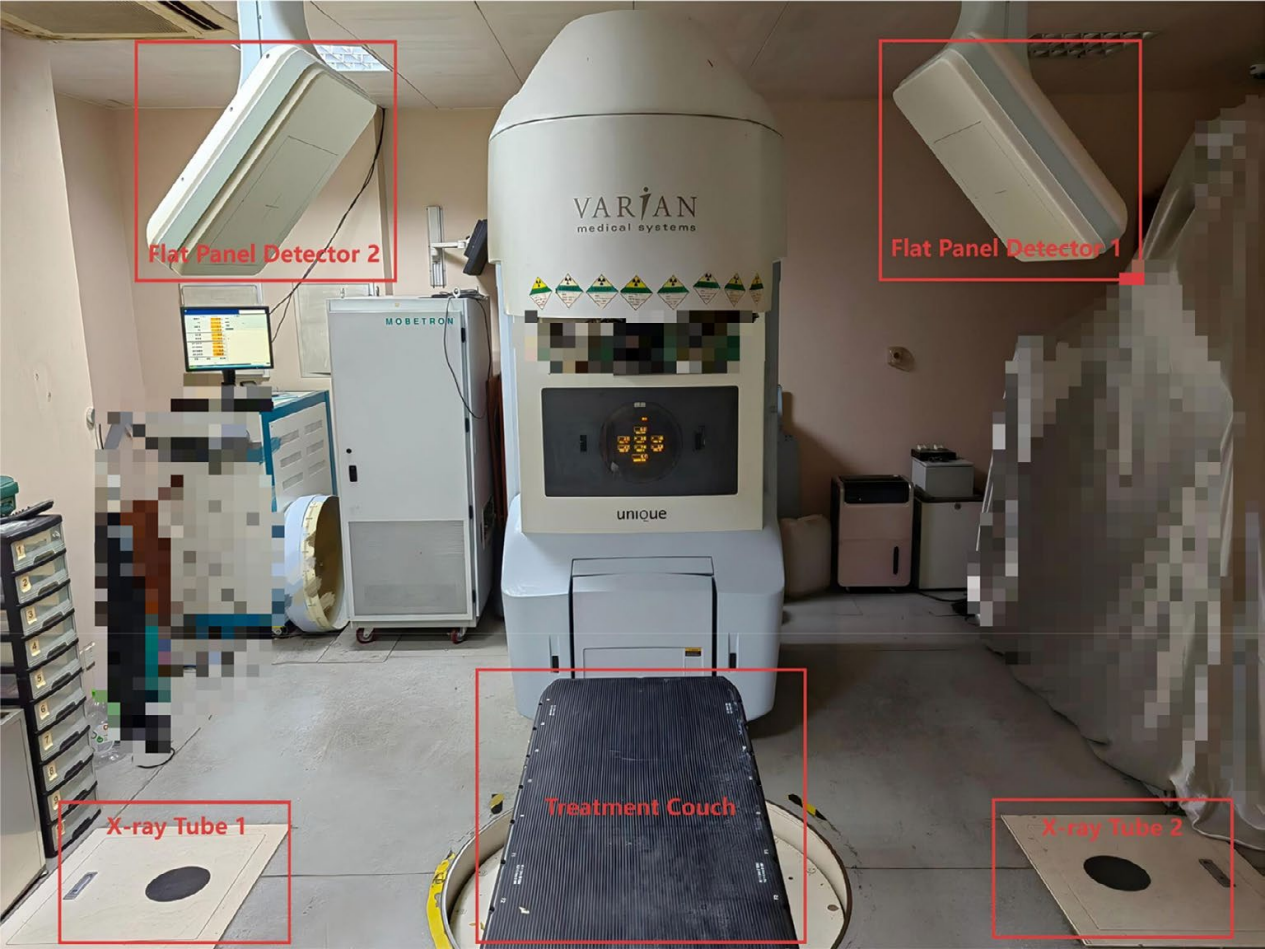
Equipment layout diagram of iSCOUT.

Before the first treatment, the therapist obtained an EPID verification image at gantry angles of 0° and 270° (or 90°), with image parameters of 20 cm × 20 cm and a single exposure of 6MU, followed by an iSCOUT scan. Figure 2 shows the equipment layout diagram of iSCOUT. Portal images and iSCOUT scan images were registered with digitally reconstructed radiographs transferred from the treatment planning system (TPS) and manually fine‐tuned by the therapist. The registration results of the EPID images with the digitally reconstructed radiographs images were used as the actual treatment standard to obtain the setup error data in three directions: left–right (LR, *X*), superior–inferior (SI, *Y*) and anterior–posterior (AP, *Z*) for EPID and iSCOUT. The reference landmarks for position correction were the bony landmarks in the patient's pelvic region. For most patients, the frequency of EPID position verification was conducted once daily during the first week of treatment and then once a week thereafter. Two radiotherapy therapists confirmed the setup errors online and recorded the data, which were reviewed offline by an attending physician.

### 2.4 Statistical Analysis

Data were analyzed using the SPSS software (version 27.0). Categorical data were expressed as frequency (*n*) and percentage (%), and group comparisons were made using chi‐square and Fisher's exact tests. Continuous data were expressed as mean ± standard deviation. Group comparisons were made using analysis of variance and the Kruskal–Wallis *H* test. Since the setup errors in each direction in this study did not follow a normal distribution, they were expressed as the median [25th–75th percentile]. Group comparisons were made using the Kruskal–Wallis *H* test with Bonferroni correction for *p*‐values. Measurement differences between the EPID and iSCOUT groups were analyzed using a paired Wilcoxon test. The correlation and consistency of the error data between the EPID and iSCOUT groups were analyzed using the Spearman correlation coefficient and Bland–Altman plot, respectively. Statistical significance was set at *p* < 0.05.

Setup errors consist of systematic errors (Σ) and random errors (σ). The overall mean represented the systematic error, which was calculated as the average of the mean values for all individual patients. Σ is defined as the standard deviation of individual means and σ is the root mean square of the standard deviations of all patients [18, 19]. The calculation method is as follows: (1) For each patient, record all the setup errors (in *X*, *Y* and *Z* directions) and the total number of CBCT scans. (2) For each patient, calculate the mean setup error across all treatment fractions, denoted as the individual mean error $m_{k}$. (3) For each patient, calculate the standard deviation of setup errors across all treatment fractions, denoted as the individual standard deviation $S_{K}$. (4) Compute the weighted average of the individual mean errors *M*($m_{k}$) by multiplying each patient's mean error $m_{k}$ by the number of fractions $N_{k}$, summing across all patients and dividing by the total number of treatment fractions. (5) To calculate the weighted standard deviation of the individual mean setup errors Σ($m_{k}$), perform the following steps:

1. For each patient, compute the squared deviation from the global weighted mean ${\left(m_{k}-M\left(m_{k}\right)\right)}^{2}$, multiplied by the number of fractions $N_{k}$;
2. Sum across all patients;
3. Divide by the total number of fractions Σ$N_{k}$;
4.
$$\Sigma \left(m_{k}\right)=\sqrt{\frac{\Sigma_{K}\left({\left(m_{k}-M\left(m_{k}\right)\right)}^{2}\cdot N_{k}\right)}{\Sigma N_{k}}}.$$

1. Multiply each patient's squared standard deviation by their degrees of freedom: $S_{K}^{2}\cdot$ ($N_{K}-1$);
2. Sum across all patients;
3. Divide by the total degrees of freedom Σ($N_{K}-1$);
4. RMS ($S_{K}$) =$\;\sqrt{\frac{\Sigma_{K}\left(S_{K}^{2}\cdot \left(N_{K}-1\right)\right)}{\Sigma_{K}\left(N_{K}-1\right)}}$.

(6) To calculate the root mean square (RMS) of the individual standard deviations $S_{K}$ for each patient (which reflects the overall random setup error), perform the following steps:

*M* refers to $M\left(m_{k}\right)$, the weighted arithmetic mean of individual mean setup errors, indicating the overall systematic deviation; Σ refers to Σ($m_{k}$), the weighted standard deviation of individual mean errors, representing the magnitude of systematic error; *σ* refers to RMS ($S_{K}$), the root mean square of individual standard deviations, reflecting the overall random error.

The margin from the clinical target volume to the planning target volume (PTV) was calculated using the formula proposed by van Herk et al. [20, 21]:

PTVmargin=2.5Σ+0.7σ.

## 3 Results

### 3.1 Distribution of Setup Errors and Group Comparisons for Different Rectal Cancer Segments

**FIGURE 3:**
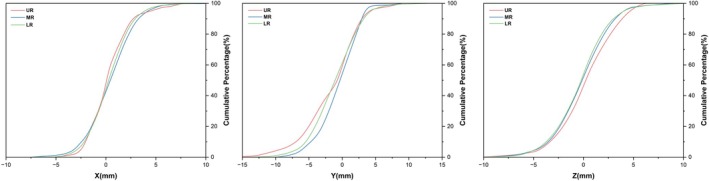
Cumulative percentage diagram of setup error range in three directions for three groups of patients.

Figure 3 shows the cumulative distribution function curves of the setup errors in the *X* and *Z* directions being relatively consistent across the three groups and rapidly increasing symmetrically around zero. Over 85% of the setup errors in the *X* and *Z* directions were within ±3 mm. In the *Z* direction, the UR group showed a trend of positive displacement compared with the other two groups. In the *Y* direction, the cumulative distribution function curves of the three groups showed different distribution trends. The proportion of errors of > 3 mm in the negative direction was higher in the UR group (35.0%) than in the MR (20.2%) and LR (30.0%) groups.

**FIGURE 4:**
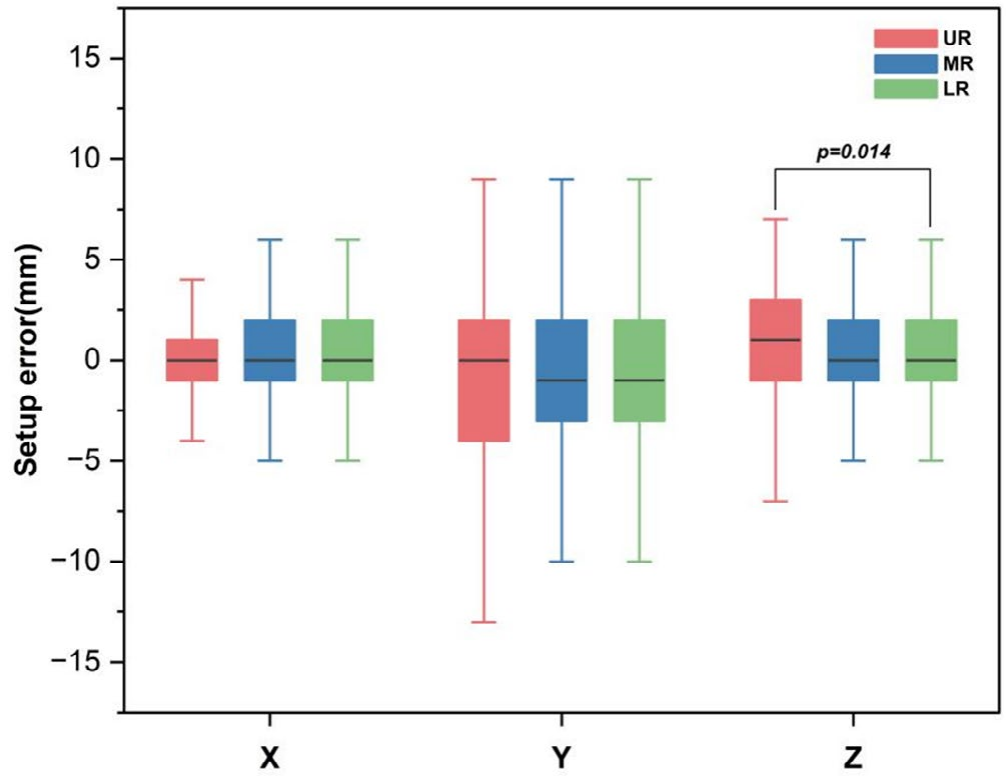
Comparison of differences in three‐dimensional directions setup errors among three groups of patients.

Table 2 shows the setup errors in the three‐dimensional directions for different rectal cancer segments. No statistically significant differences were observed among the UR, MR and LR groups in the *X* and *Y* directions. In the *Z* direction, the median [25th–75th percentile] setup errors were 1 [−1, 3] mm for UR, 0 [−1, 2] mm for MR and 0 [−1, 2] mm for LR. Post hoc tests revealed a significant difference between UR and LR (*p* = 0.014), while no significant difference was observed between UR and MR or MR and LR. Figure 4 shows more details on the data distribution.

**TABLE 2:** Comparison of setup errors between the three groups (median [25th–75th percentile], mm).

	UR	MR	LR	H	*p*
*N*	203	1022	1218	—	—
*X*	0 (−1, 1)	0 (−1, 2)	0 (−1, 2)	2.620	0.270
*Y*	0 (−4, 2)	−1 (−4, 2)	−1 (−3, 2)	1.765	0.414
*Z*	1 (−1, 3)	0 (−1, 2)	0 (−1, 2)	8.579	0.014^a^ (UR vs. LR)

*Note: N*: EPID scans. Pairwise comparisons were conducted only for directions with significant Kruskal–Wallis test results (*p* < 0.05).

Abbreviations: LR, lower rectal cancer; MR, middle rectal cancer; UR, upper rectal cancer.

^a^ The Bonferroni correction was applied.

### 3.2 Comparison of Target Margin Expansion for Different Rectal Cancer Segments

Table 3 shows the margin expansions in the three directions for the different rectal cancer segments. The UR group had the largest systematic and random errors in the *Y* direction, requiring a margin expansion of 0.83 cm. However, the other segments required 0.7 cm. Middle rectal cancer required the largest margin expansion in the *X* and *Z* directions, of 0.5 and 0.59 cm, respectively. Compared with the other two groups, the lower rectal cancer group required smaller margin expansions in all three directions.

**TABLE 3:** The PTV margin required for different segments of rectal cancer in *X*, *Y* and *Z* directions.

	UR			MR			LR		
	*X*	*Y*	*Z*	*X*	*Y*	*Z*	*X*	*Y*	*Z*
Systematic error (Σ)	0.13	0.24	0.16	0.15	0.19	0.17	0.12	0.19	0.15
Random error (*σ*)	0.17	0.35	0.24	0.19	0.30	0.23	0.17	0.31	0.23
PTV margin (cm)	0.44	0.83	0.57	0.50	0.69	0.59	0.43	0.68	0.53

Abbreviations: LR, lower rectal cancer; MR, middle rectal cancer; PTV, planning target volume; UR; upper rectal cancer.

### 3.3 Measurement Differences Between EPID and iSCOUT

**FIGURE 5:**
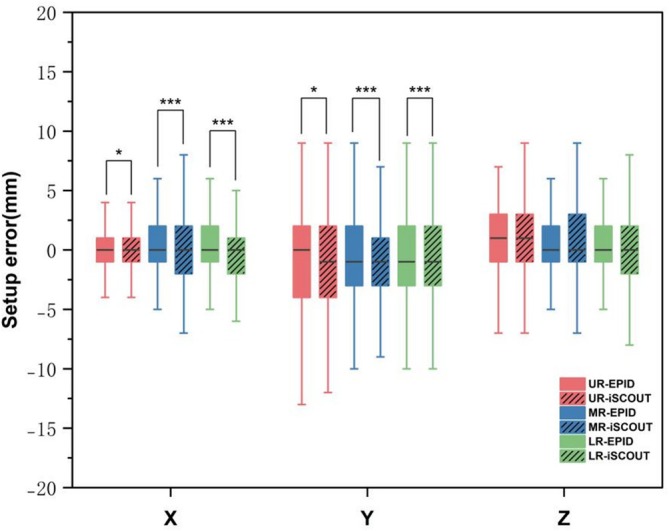
Comparative analysis of EPID and iSCOUT setup errors data across rectal cancer segments.

Table 4 shows the measurement data based on the two image‐guided methods: EPID and iSCOUT. Significant differences were observed in the *X* and *Y* directions among the UR, MR and LR groups. In the *Y* direction, the mean setup errors measured by EPID for the UR, MR and LR groups were −1.12, −0.77 and −0.60 mm, respectively, compared with −1.52, −1.04 and −0.82 mm measured by iSCOUT, while in the *Z* direction, the corresponding mean values measured by EPID were 0.75, 0.34 and 0.22 mm, compared with 0.95, 0.39 and 0.24 mm by iSCOUT, respectively. The EPID group had higher values in the *X* direction than the iSCOUT group (0.33 vs. 0.02 mm, 0.44 vs. 0.09 mm, 0.37 vs. −0.01 mm). Figure 5 shows the data distribution.

**TABLE 4:** Comparison of setup errors between EPID and iSCOUT in different directions and segments (median [25th–75th percentile], mm).

	UR			MR			LR		
	*X*	*Y*	*Z*	*X*	*Y*	*Z*	*X*	*Y*	*Z*
EPID	0 (−1, 1)	0 (−4, 2)	1 (−1, 3)	0 (−1, 2)	−1 (−3, 2)	0 (−1, 2)	0 (−1, 2)	−1 (−3, 2)	0 (−1, 2)
iSCOUT	0 (−1, 1)	‐1 (−4, 2)	1 (−1, 3)	0 (−2, 2)	−1 (−3, 1)	0 (−1, 3)	0 (−2, 1)	−1 (−3, 2)	0 (−2, 2)
*Z*	−2.385	−2.815	−1.770	−5.443	−5.443	−1.005	−6.832	−5.084	−0.460
*p*	0.017	0.005	0.077	0.001	0.001	0.315	0.001	0.001	0.646

Abbreviation: EPID, electronic portal imaging device.

### 3.4 Correlation Analysis of Measurement Results Between EPID and iSCOUT

**FIGURE 6:**
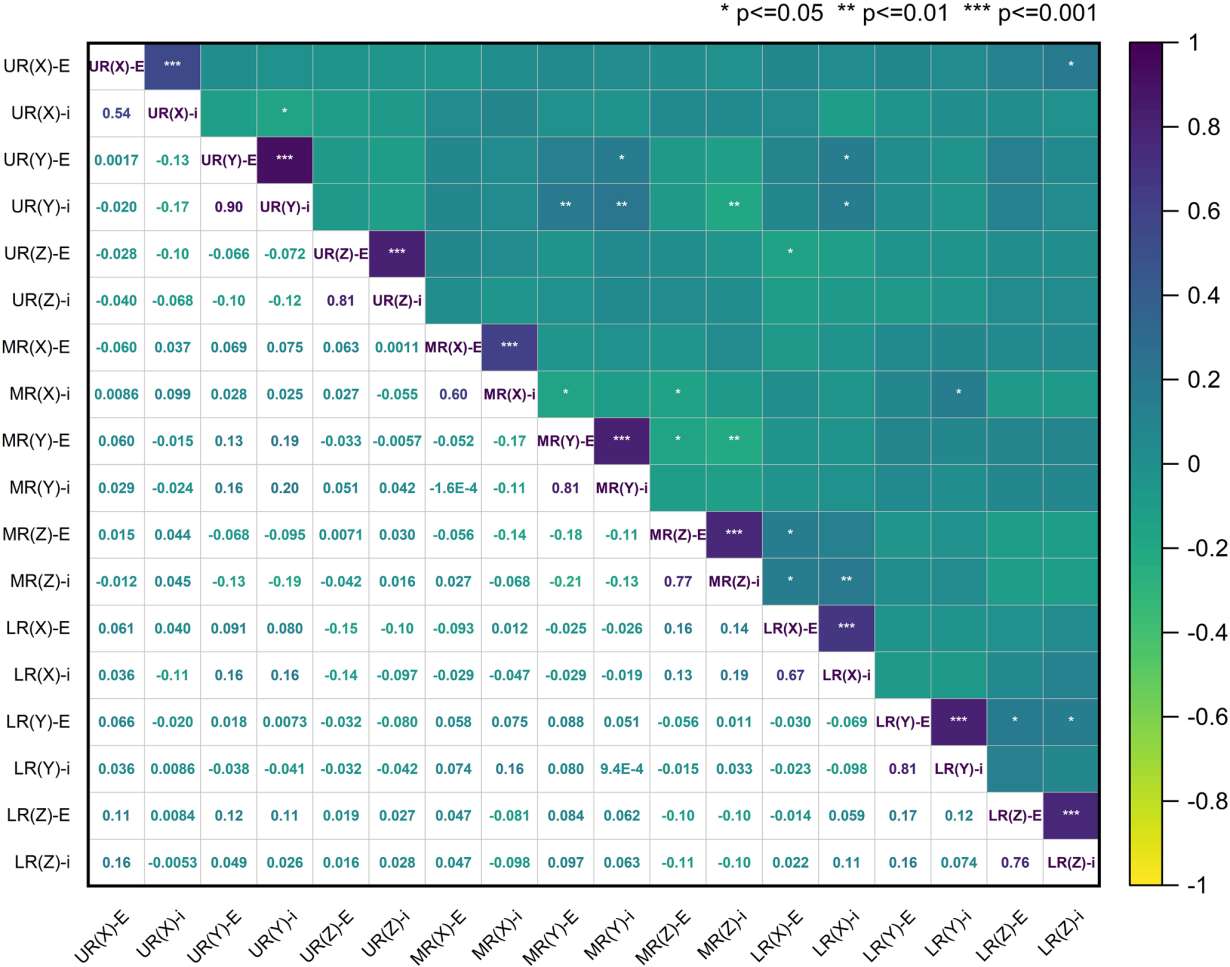
Correlation analysis of EPID and iSCOUT setup errors data across rectal cancer segments.

Figure 6 shows the correlation analysis results of setup errors for different rectal cancer segments along the *X*, *Y* and *Z* directions measured by EPID and iSCOUT‐guided methods. The registration results of EPID in all directions for different rectal cancer segments were significantly correlated with the corresponding iSCOUT error data (*p* < 0.001). For the UR, MR and LR groups, strong and moderate correlations between the setup errors measured by EPID and iSCOUT were observed in the *Y* and *Z* directions, with correlation coefficients (*r*) values of 0.90 and 0.81 for the UR group, 0.81 and 0.77 for the MR group, and 0.81 and 0.76 for the LR group, respectively. In the *X* direction, the setup errors obtained from EPID and iSCOUT also showed moderate correlations, which were lower than those in the *Y* and *Z* directions. Specifically, the *r* values were 0.54, 0.60 and 0.67 for the UR, MR and LR groups, respectively.

### 3.5 Consistency Analysis of Measurement Results Between EPID and iSCOUT

**FIGURE 7:**
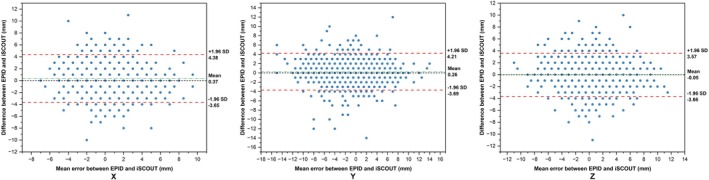
Consistency analysis of EPID and iSCOUT setup errors data.

The 95% limits of agreement (LoA) between EPID and iSCOUT measurements in the *X*, *Y* and *Z* directions were −3.65 to 4.38, −3.69 to 4.21 and −3.66 to 3.57 mm, respectively (Figure 7). The mean lines in the *X* and *Y* directions were 0.37 and 0.26 mm, respectively. The Bland–Altman plot showed that some outliers exceeded the limits of agreement and were randomly distributed. The percentages of data points within the 95% LoA in the *X*, *Y* and *Z* directions were 93.5%, 95.3% and 93.9%, respectively.

## 4 Discussion

In this study, we aimed to quantify the setup errors associated with different rectal cancer segments and analyze the differences between the groups. The results showed that in the cumulative percentage plots of setup errors across three directions for different rectal cancer segments, 85% of the error data in the *X* and *Z* directions were within ±3 mm for all three groups, indicating good positional consistency between the groups. The error trends in the *Y* direction were inconsistent among different rectal cancer segments, and extreme values of setup errors > 5 mm were observed in the negative direction in all groups. This may be related to changes in the surface positioning lines caused by skin traction during patient setup by therapists, as reported by Xu et al. [15]. In the *Z* direction, the upper rectum group showed a trend of positive displacement compared with the other two groups, suggesting a certain systematic bias in this direction, which was significantly different from that in the lower rectum (*p* = 0.013). These findings indicate that patients in different anatomical subgroups exhibit distinct displacement characteristics along the *Z*‐axis. Previous CBCT‐based studies comparing different rectal segments in a cohort of 16 patients have similarly demonstrated greater morphological and positional variations in the upper rectum compared with the lower rectum [22]. Additionally, changes in patient‐related factors, including body‐mass factor (BMF) during treatment, may cause error displacement [16, 17].

An appropriate PTV margin is clinically valuable for improving the accuracy of intensity‐modulated radiotherapy for rectal cancer. It ensures adequate target dose coverage while limiting the dose to organs at risk [23, 24]. In this study, we calculated the required margin expansion for different segments of rectal cancer. The margin expansion in the *Y* direction was higher than that in the other directions, and at least 0.83 cm was required for upper rectal cancer to meet clinical needs. Tamponi et al. [25] reported that patients with prostate, rectal and gynecological tumors undergoing radiotherapy required a clinical target volume‐PTV margin expansion of 1 cm in the *X* direction and 2 cm in the *Y* direction. In comparison, our study also showed that a larger margin expansion was required in the *Y* direction, but the required expansion was < 1 cm. Thasanthan et al. [26] reported 50 patients with rectal cancer with the largest random error in the *Y* direction at 0.33 cm. They suggested margin expansions of 0.76, 0.93 and 0.84 cm in the *X*, *Y* and *Z* directions, respectively, requiring a larger margin expansion in the *Y* direction. This result aligns with our study results, with random errors in the *Y* direction exceeding 0.3 cm for different rectal cancer segments, requiring margin expansions of 0.83, 0.69 and 0.68 cm, respectively.

Currently, IGRT is commonly used in clinical practice to ensure consistency between the patient's position during CT localisation [27]. Common IGRT techniques include EPID and cone‐beam CT. Moreover, iSCOUT provides extremely short exposure times and relatively low imaging doses per acquisition. When comparing the error data obtained from the EPID and iSCOUT technologies, statistically significant differences were observed in the *X* and *Y* directions. Certain systematic biases exist between the two devices in the *X* and *Y* directions, which need to be corrected. The correlation heatmap showed a strong and moderate correlation in the same direction within the same group (*p* ≤ 0.001). The Bland–Altman plot showed the mean lines in the *X* and *Y* directions as 0.37 and 0.26 mm, respectively, indicating a small systematic bias between the two measurement results in this direction. This further confirms the results of the paired test between them. The percentages of data points within the 95% LoA were 93.5%, 95.3% and 93.9%, respectively, indicating good overall consistency. There were statistically significant differences in the *X* and *Y* directions; however, the average difference was small (0.3 mm), and the 95% LoA coverage was nearly 95%, indicating that the iSCOUT image‐guided patient setup system is mature and reliable, serving as an alternative image‐guided technology when necessary.

This study has some limitations. First, owing to the registration characteristics of the EPID, we were unable to study the displacement of soft tissues, including the rectum and bladder, between the fractions. Second, this study did not consider the impact of changes in patient weight or abdominal circumference on setup errors. Since changes in patient weight during treatment and the uncertainty of body mass factor are important factors affecting setup errors [28, 29], these variables will be the focus of future studies.

## Author Contributions

**Bao Wan:** data curation, formal analysis, methodology, software, visualisation, writing – original draft. **Jiangtao Han:** data curation, formal analysis, methodology, software, visualisation, writing – original draft. **Qian Wu:** data curation, formal analysis, writing – review and editing. **Jingjing Lu:** investigation, writing – review and editing. **Xueshan Li:** investigation, writing – review and editing. **Yongtai Zheng:** investigation, writing – review and editing. **Chao Liu:** investigation, writing – review and editing. **Shuo Sun:** investigation, writing – review and editing. **Yu Zhao:** investigation, writing – review and editing. **Fukui Huan:** investigation, writing – review and editing. **Tantan Li:** conceptualisation, investigation, supervision, writing – review and editing.

## Funding

This work was supported by CAMS Innovation Fund for Medical Sciences (CIFMS) (2025‐I2M‐C&T‐B‐050).

## Ethics Statement

This study was performed at the Cancer Hospital, Chinese Academy of Medical Science, and approved by the hospital's ethics committee (ethical approval number: 23/049‐3788).

## Consent

All patients provided written informed consent.

## Conflicts of Interest

The authors declare no conflicts of interest.

## Data Availability

The data that support the findings of this study are available from the corresponding author upon reasonable request.
